# Neurophysiological outcomes of combined transcranial and peripheral electromagnetic stimulation on DOMS among young athletes: A randomized controlled trial

**DOI:** 10.1371/journal.pone.0312960

**Published:** 2025-07-08

**Authors:** Hugo Kériven, Alberto Sánchez-Sierra, Ángel González-de-la-Flor, María García-Arrabé, María Bravo-Aguilar, Marta de-la-Plaza-San-Frutos, Guillermo García-Pérez-de-Sevilla, José Francisco Tornero Aguilera, Vicente Javier Clemente-Suárez, Diego Domínguez-Balmaseda

**Affiliations:** 1 Department of Real Madrid Graduate School, Faculty of Medicine, Health and Sports, Universidad Europea de Madrid, Madrid, Spain; 2 Department of Physiotherapy, Faculty of Medicine, Health and Sports, Universidad Europea de Madrid, Madrid, Spain; 3 Faculty of Phisioterapy and Nursing, Universidad de Castilla-La Mancha, Toledo, Spain; 4 Toledo Physiotherapy Research Group (GIFTO), Faculty of Phisioterapy and Nursing, Universidad de Castilla-La Mancha, Toledo, Spain; 5 Research Group on Exercise Therapy and Functional Rehabilitation, Faculty of Health Sciences, Universidad Europea de Madrid, Madrid, Spain; 6 Department of Sports Sciences Faculty of Medicine, Health and Sports, Universidad Europea de Madrid, Madrid, Spain; 7 Masmicrobiota Group, Faculty of Health Sciences, Universidad Europea de Madrid, Madrid, Spain; Università degli Studi di Milano: Universita degli Studi di Milano, ITALY

## Abstract

This study investigates the potential benefits of a combined electromagnetic stimulation therapy, involving both transcranial and peripheral stimulation (paired-associative electromagnetic stimulation), to address Delayed Onset Muscle Soreness (DOMS). Forty-eight young athletes participated in this randomized controlled trial and were allocated to the control group (n = 12), the peripheral group (n = 13), the transcranial group (n = 11), and the combined group (n = 12). Surface electromyography (EMG) during leg extension and peak force were used to assess the response of the peripheral nerves. Additionally, force dynamometry and the Counter Movement Jump (CMJ) test were employed to evaluate the progression of lower limb sports performance over the study period. All assessments were performed before and after the eccentric exercise session that induced DOMS, as well as at 24-, 48-, and 72-hours post-exercise. The combined group exhibited significantly greater muscle activation in both electromyographic recordings compared to the other groups (p < 0.001), with large effect sizes for EMG peak in vastus medialis (η²p = 0.786), vastus lateralis (η²p = 0.821), and rectus femoris (η²p = 0.816). Moreover, the combined group demonstrated a marked improvement in both force dynamometry (η²p = 0.593) and CMJ performance (η²p = 0.520), with significant differences observed compared to the other groups (p < 0.001). In conclusion, paired-associative electromagnetic stimulation shows promise in enhancing muscle activity and improving lower limb performance by facilitating recovery from DOMS in young athletes. The study was registered with the Australian New Zealand Clinical Trials Registry (ACTRN12623000677606) on June 23^rd^, 2024 (https://anzctr.org.au/).

## Introduction

Over the past decades, Delayed Onset Muscle Soreness (DOMS) has been increasingly recognized as a potential precursor to more severe athletic ailments. DOMS may be linked to alterations in nerve excitability, an insight that offers opportunities for further exploration [1].

When damage occurs within the muscle spindle, an initial chemoreceptor, Piezo 2, is activated. The primary role of Piezo 2 is to protect neurons. By remaining inactive, it is believed to protect the nerve; however, it is theorized that strenuous exercise may impair this protective mechanism. Prolonged stress on the muscle may consequently damage the nerve and affect its conduction capacity. Moreover, it has been demonstrated that the Piezo 2 receptor is directly linked to the hippocampus, suggesting a potential connection between DOMS and the central nervous system [2]. On top of that, therapies focused on either peripheral or central stimulation could tigger the Piezo 2 chemoreceptor by relaxing and taking down the stress part of the strenuous activity which could decrease the expression of the symptoms by the patients [3,4].

The manifestation of DOMS is commonly observed when athletes resume intensive physical activity after a period of rest. This type of micro-damage typically occurs when individuals perform unusual eccentric exercises or tasks. Even for trained athletes, as in the present study, a prolonged break from the main activity could exacerbate the occurrence of DOMS upon resumption of activity [5]. Despite their high level of physiological adaptation, conditioning trainers in sports often associate the pre-season period with an increased likelihood of DOMS, alongside a higher probability of injury risk [6].

This condition is often accompanied by the involvement of various biochemical substances and factors signalling its onset [7]. Among these, the secretion of bradykinin through sweat during physical exertion and the increase in nerve growth factor are noteworthy. These elements are implicated in the sensitization of nociceptors, potentially exacerbating the perception of soreness [8].

A novel perspective in the understanding of DOMS focuses on induced hyperalgesia resulting from muscle fiber damage. This theory postulates that DOMS might arise from axonal compression at the neuromuscular spindle level, leading to two outcomes: acute muscle compression and excitotoxicity due to glutamate release [9]. Consequently, athletes experience heightened sensitivity and a noticeable decrease in strength [10].

Expanding on this hypothesis, DOMS may result from acute axonopathy, which also triggers inflammation in the surrounding tissues. This condition is characterized by a biphasic process: the initial damage phase, where high-intensity muscular work harms the neuromuscular spindle, and the subsequent phase, which includes observable signs such as inflammation, pain, stiffness, decreased strength, and reduced electrical activity in the peripheral nerve [11]. DOMS is a complex phenomenon that not only results from muscle fatigue but also involves acute damage to muscle fibers, which triggers biochemical responses such as bradykinin release and upregulation of nerve growth factor, enhancing the sensitivity of nociceptors [12–16]. This multifaceted mechanism suggests that both the peripheral and central nervous systems play crucial roles in the manifestation of DOMS. Recent research has started to explore not only the isolated effects of each nervous system but also the interaction between them, which may be of particular interest in understanding how conditions like DOMS could influence both systems simultaneously. Studies have demonstrated that electromagnetic stimulation can target both the central and peripheral nervous systems, offering potential therapeutic benefits by modulating these pathways. The combined effects of such stimulation could not only mitigate pain but also improve recovery and performance outcomes, as previous studies have highlighted the significant impact of these interventions on both neural systems [12,16]. Thus, understanding how these systems interact during DOMS is crucial for developing more effective recovery strategies.

At the central nervous system level, the motor evoked potential (MEP), which is an electrical potential registered from a muscle during transcranial magnetic stimulation (TMS), provides further insight. It has been shown that TMS can assess central resistance to fatigue by measuring MEP latency, defined as the time required for the central signal to reach the muscle [17]. TMS has revolutionized the investigation of cortical excitability through its non-invasive approach [16], and its scope has extended beyond medical applications to include conditions such as fibromyalgia and post-stroke rehabilitation [18]. Other authors have explored the influence of sports activities on brain excitation, particularly its correlation with muscle fatigue [19]. This line of inquiry is crucial for understanding the central nervous system’s response to exercise and its role in muscle relaxation following eccentric contractions that induce DOMS [20].

Moreover, the targeted application of TMS offers potential therapeutic benefits in sports science, such as promoting muscle relaxation by modulating muscle activation patterns [21]. The Primary Motor Cortex (M1), which is deeply involved in voluntary skeletal muscle contraction, has demonstrated a high capacity for adaptation through motor practice, resulting in observable changes. In the cases studied, motor improvement was accompanied by an increase in motor evoked potentials (MEP), enhanced corticospinal responses, and further insight into the cortico-spinal tract, obtained through TMS stimulation, which provides a deeper understanding of its influence on skeletal muscle activity [17].

Another non-invasive therapy, Peripheral Electromagnetic Stimulation (PES), emerges as a complementary strategy. PES delivers rapid pulses of high-intensity electricity combined with a magnetic field. Unlike TMS, PES primarily targets peripheral regions of the body [22]. PES activates peripheral afferents when applied to muscles, thereby influencing brain activation and neuroplasticity. Such stimulation could indirectly activate mechanoreceptors in muscle fibers, affecting the athlete’s sensory processing and motor control. When applied over the M1 area, it could be valuable to assess muscle response using electromyography (EMG) to observe the combined treatment’s capacity to restore normal muscle activation [23].

Therefore, the fatigue phenomenon is directly linked with the perturbation over the nerve signal as long as the thoughts that influences the potential outcome. As the Long-Term Potentiation (LTP) protocol applied by the PES over 5 minutes which could normalized the nerve signal as and the TMS application that already demonstrated having impacts on the muscle strength and function the present combination of both stimulation could have positives impacts on the DOMS conditions and its associates factors as previous study had demonstrated [3,17].

Recent research suggests potential benefits of a combined therapy. This integrative approach aims to mitigate exercise-induced fatigue at both central and peripheral levels by modulating cortical excitability [24]. The current study aimed to investigate the effectiveness of the combined application of TMS and PES on recovery in young athletes over the 72-hour period following the induction of DOMS.

## Materials and methods

### Study design and setting

This study was a randomized, controlled, double-blind trial involving young athletes. It followed the Helsinki ethical guidelines [25] and adhered to the Consolidated Standards of Reporting Trials (CONSORT) guidelines. All participants received a verbal explanation of the study procedures and completed a written informed consent form before beginning the study.

The study was approved by the Research Ethics Committee of the Hospital Clínico San Carlos (Madrid, Spain) (ref. number: C.I.23/048-F). All participants provided informed consent to take part in the study. Additionally, the study was registered with the Australian New Zealand Clinical Trials Registry (ACTRN12623000677606) on June 23^rd^, 2024 (https://anzctr.org.au/).

Recruitment took place from July 1st to July 30th, 2023. Participants were randomly assigned to one of four groups: the Control group (Cont), which received no intervention; the Super Induction group (P); the Transcranial group (T); and the Combination Stimulation group (Comb). Treatments were administered at a neutral location, and researchers assigned to treatment stations were solely responsible for delivering the interventions.

### Sample size calculation

The sample size calculation was performed using G*Power software (version 3.1). An F-test for an ANOVA with repeated measures, accounting for within-group and between-group interactions, was chosen. Input parameters included a medium effect size (f = 0.20), a significance level of 0.05, a power of 0.8, four groups, five measurements, with a correlation among repeated measurements of 0.5 and a non-sphericity correction of 1. The calculation indicated that a total sample size of 48 participants would be required to achieve sufficient statistical power. To account for potential attrition (10%), the sample size was increased to 52 participants (13 per group) to compensate for possible dropouts.

### Participants

The research was conducted with participants from a university, selected based on specific inclusion and exclusion criteria to ensure the relevance of the study results. Recruitment efforts were made through communication channels and strategically placed advertisements within the university’s Faculty of Sports Sciences.

Inclusion criteria included males aged 18–35 years, engaged in regular physical activity at least three times per week for a minimum of one year, and exhibiting no hypersensitivity in areas to be treated with peripheral stimulation. Exclusion criteria were a diagnosis of chronic disease, a musculoskeletal injury to the lower extremities in the previous six months, and smokers. Only males were included in this study, based on the work by Chen et al. (2019), which highlighted that both creatine kinase and lactate concentrations tend to differ between males and females performing similar athletic activities [26]. As highlighted in previous work in scientific literature, the female athletes could experience fluctuations at the hormonal level specially the estrogen and progesterone level which could influence various parameters such as: muscle strength and power output, neuromuscular control assed with EMG signal, the perception of soreness and pain threshold. All of the present potential variation within the previous parameters could complicate the interpretation of recovery data, especially studying during a DOMS process [27,28]. As evocated before, male and female experience a DOMS process in different ways both in term of subjective soreness and objective recovery markers (e.g.,: CK concentration) which could miss lead potential findings in the present study [29,30].

**Fig 1:**
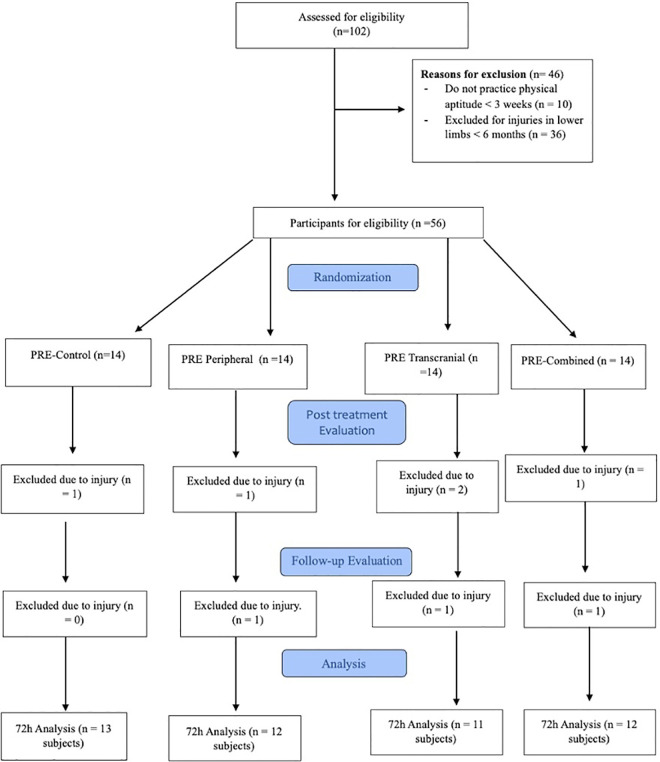
Flowchart of the study following the CONSORT regulations.

A total of 102 volunteers consented to participate in this study. However, before the initial assessment, 46 individuals were unable to partake in the study, and an additional 8 participants were unable to complete all the required evaluations, because they suffered from an injury in the lower limb which is incompatible with the requirement of the study. Consequently, 48 male athletes were included in the final analysis, with an average age of 21.95 ± 4.23 years and a Body Mass Index of 23.27 ± 2.41 kg/m^2^ (Fig 1).

### Randomization

Group randomization was performed using the randomization function in Microsoft Excel (Microsoft Corporation, Redmond, Washington, USA). Participants were randomly assigned to one of the four study groups as previously described. The double-blind design ensured that neither the examiners nor the data analysts were aware of participants’ group allocations during the study. A designated room was set up for participants to receive their treatments, maintaining the blinding protocol. Each participant was assigned a number to record the study data, ensuring that no member of the research team could identify the group to which they had been assigned.

### Procedure

**Fig 2:**
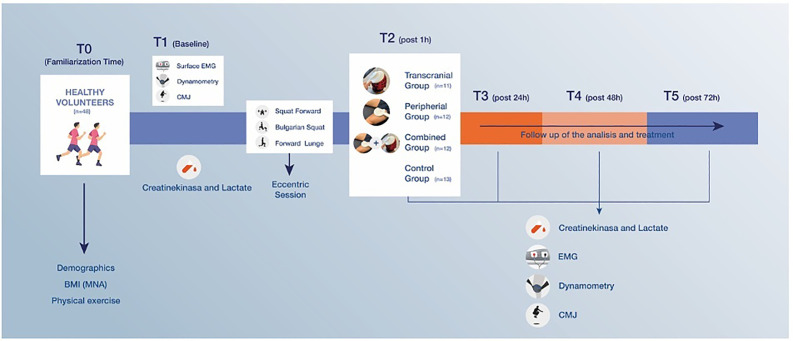
Overview of the Assessment Timeline and Intervention Protocol.

The study’s methodology involved a structured series of five assessment sessions for each participant (Fig 2). A familiarization session was conducted one week before the first assessment to acquaint participants with the procedures and equipment.

The first assessment session (T1) was comprehensive, including a range of physiological and biomechanical measurements. These measurements encompassed creatine kinase (CK) levels, blood lactate concentration, performance in the Counter Movement Jump (CMJ), quadriceps dynamometry, surface electromyography (EMG) of the quadriceps, and anthropometric data. This broad array of measurements was chosen to provide a holistic understanding of the participants’ baseline physiological and physical states.

Subsequent sessions were scheduled at specific intervals post-exercise—namely, 1 hour (T2), 24 hours (T3), 48 hours (T4), and 72 hours (T5) after the induction of muscle damage—to monitor progression and recovery over time (Fig 2).

The evaluation of muscle damage primarily focused on the analysis of blood creatine kinase (CK) and lactate concentrations. These were obtained from pinprick blood samples and analyzed using electrophoretic analysis (Lactate Scout Pro, Musimedic S.L, Donostia, Spain). To ensure the accuracy of these enzyme measurements, participants were instructed to abstain from any physical activity for at least two days prior to the study, before the baseline assessment (T1). After completing the first day of the study (including the T1 and T2 assessments), participants were allowed to resume normal physical activity while completing the remaining study timeline (T3 to T5).

### Intervention

#### Eccentric exercise protocol (Fig 3).

The exercise session was designed in three distinct phases:

**Fig 3:**
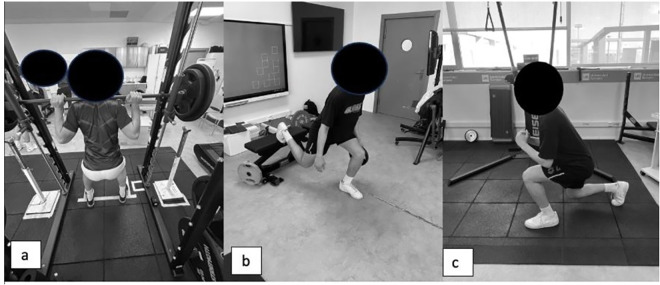
Illustration of participants engaged in the eccentric exercise protocol. Exercise Demonstrations Include: a) Front Squat; b) Bulgarian Squat; c) Frontal Stride.

- General Warm-Up: The initial phase involved a warm-up focused on enhancing joint mobility in the lower limbs and performing bodyweight strength exercises. This preparatory phase was aimed at conditioning the athletes for the subsequent exercises intended to induce DOMS.
- Intervention Exercises: Participants engaged in a series of three exercises. The central component of this phase was the encoder-controlled squat exercise. Squat execution was quantified using a linear accelerometer, calibrated to measure 60% of the participants’ one-repetition maximum (1-RM) [31].
- Eccentric Workout Routine: The final phase included the following three exercises: a) Squat Forward: Participants performed 10 sets of 10 repetitions at 60% of their 1-RM, as determined during the pre-study workout session. The protocol was based on the study by Pearcy et al. (2015), where DOMS was induced by 10 sets of 10 repetitions of Squat Forward at 60% of 1-RM, with 4 seconds (s) of eccentric contraction followed by 1s of concentric contraction, and a 2-minute rest period between sets. This exercise was chosen for the eccentric protocol because such contractions are more likely to induce DOMS compared to conventional weight training [32].
  Bulgarian Squat: This exercise involved 3 sets of 10 repetitions per leg, with the option to add 5 or 10 kg of weight.
  Forward Lunge: This exercise consisted of 3 sets of 10 repetitions per leg, with the option to add 5 or 10 kg of weight, as illustrated in Fig 3.

#### Protocol for transcranial and peripheral electromagnetic stimulation in the study.

The study employed a differentiated approach depending on the participants’ group allocation:

- Control (Cont) Group: The electromagnetic stimulation machine was positioned identically to the active treatment groups. However, the machine was turned off, and pre-recorded sounds of the machine’s operation were played during the session. For transcranial stimulation, positioning the flap corresponding to TES was considered adequate.
- Super Inductive (P) Group: Participants in this group received PES using a Long-Term Potentiation protocol to initiate recovery from DOMS with the peripheral stimulation only. This protocol involved five stimulations at 100 Hz, each lasting 5 seconds, with 55-second rest intervals [33]. The total stimulation time for this group was 10 minutes.
- Transcranial Stimulation (T) Group: This group received Transcranial Electromagnetic Stimulation (TES) consisting of 2000 pulses over at least 20 minutes, targeting the M1 cortical area (Fig 2) [34]. For TMS coil placement, the study followed the recommendations of Magrex, which provided a stimulation helmet indicating where the coil should be positioned. To ensure the correct location, as M1 is associated with voluntary contraction of skeletal muscles, particularly in the lower limbs, the minimum intensity required to generate muscle activation was calculated based on standard TMS protocol practice [20].
- Combined Stimulation (Comb) Group: Participants in this group received a combination of both PES and TES treatments, with the total stimulation time extending to 30 minutes.

In all groups, treatment began one hour after the eccentric exercise session, aligning with the onset of fatigue at T2 in the study. The duration of stimulation varied according to group assignment, ranging from 10 minutes for the P group to 30 minutes for the Comb group.

**Fig 4:**
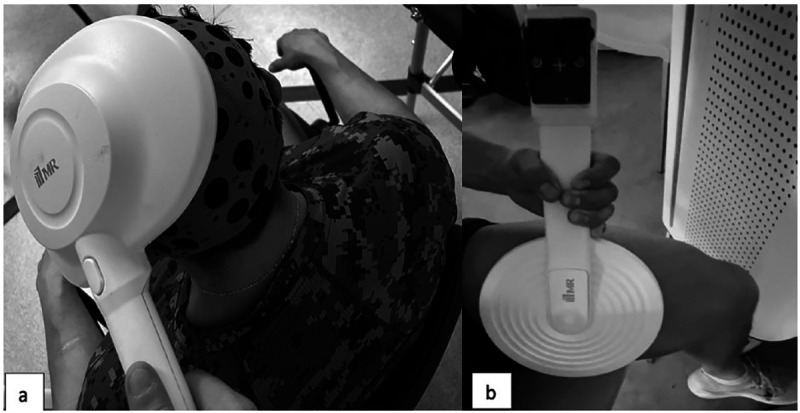
Illustration of Study Participants Undergoing Electromagnetic Stimulation: a) Transcranial Stimulation Setup; b) Peripheral Stimulation Setup.

TES and PES treatments were administered using a MagRex magnetic stimulator equipped with either a ring-shaped or 8-shaped coil (MR Inc., Republic of Korea, http://www.mrev.co.kr) (Fig 4).

### Primary outcome measure: surface electromyography

In this study, the mDurance® EMG system (mDurance Solutions SL, Granada, Spain) was used. This portable surface EMG (sEMG) system integrates three components: sensors, mobile computing, and a cloud-based data analysis framework. Statistical analysis was conducted on CK and lactate outcomes, as both variables were considered primary intervention outcomes. The primary objective was to assess the effectiveness of the DOMS protocol and its impact on participants across different groups, ensuring that the exercise protocol successfully induced muscle micro-damage. CK and lactate concentrations served as biomarkers for muscle damage in blood, and measuring these variables in each participant verified the occurrence of DOMS-related micro-damage [35,36]. First for the CK measurement using the Reflotron Plus (Roche Diagnostics, Barcelona, Spain) has shown high reliability in previous studies. According to Barid et al. (2012), the instrument used in the present study has higher inter-rater reliability and low intra-rater variability, which guarantees accuracy in the measurement of the CK levels. In addition, internal and external controls were applied to ensure the stability of the instrument during the sample collection [37].

In the case of the Lactate measurement, the Lactate Scout Pro device (Musimedic S.L, Donostia, Spain) has shown high reliability compared to other traditional methods, as evidenced in the study by Clemente et al. (2010). The reliability of the measurement was confirmed through reproducibility and precision tests, which yielded a coefficient of variation within the margins establish by international regulations. Furthermore, studies such as that of Hopkins (2000) have demonstrated the validity and reliability of electroenzymatic measurement devices in determining lactate, with a reliability coefficient greater than 95% in repeated measurements [38].

Muscle activity recorded via EMG was measured at two distinct moments: during maximal voluntary contraction (peak contraction) and during leg extension to observe potential differences between both actions.

For each recording the sensors were applied flowing the normative of the Surface ElectroMyoGraphy for the Non-Invasive Assessment of Muscles (SENIAM) to ensure a standardization across sessions, as described [39]:

First Sensor – Rectus Femoris (RF) Evaluation: Participants were positioned on a stretcher with slightly flexed knees and the trunk reclined. Electrodes were placed midway between the anterior superior iliac spine (ASIS) and the top of the patella. The ground electrode was positioned on the patella.

Second Sensor – Vastus Lateralis (VL) and Vastus Medialis (VM) Activity Recording: For VL, electrodes were placed two-thirds of the distance along the line from the ASIS to the lateral part of the patella. For VM, electrodes were positioned at 80% of the distance from the ASIS to the anterior border of the internal lateral ligament.

Surface muscle activity was captured using two bipolar sensors from Shimmer Research Ltd, Dublin, Ireland. Data recording and transmission were managed via the mDurance® mobile app, installed on a Galaxy A7 Android tablet (ZtotopCase, Suwon, South Korea) [40,41].

### Secondary outcome measures

#### Dynamometry.

Quadriceps strength was measured using the ActiveForce 2 device (Activbody, San Diego, CA). Maximal isometric quadriceps strength was assessed during knee extension with participants seated and the knee at a 90-degree angle. A motion-restricting strap was applied to the mid-third of the leg. Participants were instructed to sustain the contraction for 5 seconds, repeating the process twice with a 5-minute rest between contractions [42].

#### Counter Movement Jump (CMJ).

Participants performed the CMJ with hands on their hips, executing a knee flexion followed by rapid extension to reach maximum jump height. The CMJ was recorded using the validated My Jump 2 application, which captured each jump in slow motion at 240 frames per second using an iPad Pro 10. The tablet was consistently positioned for all recordings to ensure stable and reliable data [43].

### Statistical analysis

Statistical analyses were performed using SPSS v.29 (IBM, Armonk, NY, USA). Normal data distribution was assessed through histograms and the Shapiro-Wilk test. Variables with a p-value < 0.05 were considered non-normally distributed, while those with a p-value > 0.05 were considered normally distributed. Descriptive statistics for normally distributed variables were presented as mean ± standard deviation, while the median and interquartile range were reported for non-normally distributed variables. A one-way analysis of variance (ANOVA) was employed to compare means between groups at baseline for quantitative variables. When assumptions were met, a 5x4 (5 measurements x 4 groups) repeated measures ANOVA was performed, with Bonferroni correction for multiple comparisons. Effect sizes were expressed as partial eta squared (ηp²), categorized as small (0.01), medium (0.06), or large (0.14). The percentage change between baseline and the different measurement times (1, 24, 48, and 72 hours) was calculated using the formula: (baseline−post measurement)/baseline*100. In the post-hoc analysis, the significance level was adjusted to p < 0.0125 to account multiple comparisons.

## Results

### Baseline data

When analysing the initial demographic characteristics of the participant population, there were no significant differences between the groups at the T1 (pre-eccentric session) measurement (Table 1). The CK and blood lactate levels results showed an increase in concentrations at T2 (1h-post), T3 (24h-post), T4 (48h-post), and T5 (72h-post) in all groups (p < 0.001), without significant interactions between groups in any of the assessment moments for both biological markers which indicates an equal eccentric session impact over the study’ groups for Creatine kinase (p = 0.357) and for Lactate (p = 0.296) (Table 2).

**Table 1:** Demographics characteristics of the study’s participants.

	Total (n = 48)	P Group (n = 13)	Cont Group (n = 12)	T Group (n = 11)	Comb Group (n = 12)	P-value
Age (years)	21.95 ± 4.23	22.14 ± 5.27	21.66 ± 4.24	20.54 ± 1.43	23.00 ± 4.12	0.548
Weight (kg)	74.58 ± 8.94	72.61 ± 9.20	76.16 ± 8.59	71.72 ± 7.17	77.75 ± 8.75	0.311
Height (cm)	179.00 ± 7.31	176.38 ± 7.07	181.00 ± 6.69	178.45 ± 4.27	180.33 ± 8.95	0.399
BMI (kg/m2)	23.27 ± 2.41	23.31 ± 2.43	23.27 ± 2.51	22.53 ± 2.33	23.89 ± 2.07	0.621

**Abbreviatures**: P, super inductive group; Cont, control group; T, transcranial group; Comb, combined group; BMI, Body Mass Index.

**Table 2:** Descriptive statistics for the CK and Lactate blood levels among the study’s groups.

		T1 (baseline)	T2 (post 1h)	T3 (post 24h)	T4 (post 48h)	T5 (post 72h)	F	P value
CK (UI/L)	P (n = 13)	65.61 ± 6.22	119.30 ± 30.01	251.69 ± 48.14	153.69 ± 41.96	75.92 ± 12.06	1.104	0.357
	Cont (n = 12)	66.58 ± 6.88	112.58 ± 21.22	274.41 ± 50.91	174.66 ± 22.32	79.66 ± 13.68		
	T (n = 11)	67.00 ± 7.91	109.00 ± 17.27	222.27 ± 64.00	139.81 ± 35.40	76.54 ± 9.15		
	Comb (n = 12)	66.25 ± 8.41	124.41 ± 17.86	267.75 ± 48.45	159.41 ± 29.46	81.58 ± 12.17		
	Total (n = 48)	66.33 ± 7.14	116.54 ± 22.55	254.64 ± 54.87	157.18 ± 34.48	78.41 ± 11.79		
Lactate (mmol/L)	P (n = 13)	1.38 ± 0.17	6.03 ± 2.48	2.17 ± 0.65	3.59 ± 5.85	1.37 ± 0.25	1.271	0.296
	Cont (n = 12)	2.34 ± 3.04	7.18 ± 1.60	2.04 ± 0.65	1.65 ± 0.33	1.26 ± 0.13		
	T (n = 11)	2.43 ± 3.50	7.78 ± 6.88	2.12 ± 0.47	2.01 ± 0.57	1.50 ± 0.33		
	Comb (n = 12)	1.52 ± 0.24	8.20 ± 8.43	2.33 ± 0.57	1.92 ± 0.22	1.40 ± 0.23		
	Total (n = 48)	1.90 ± 2.24	7.26 ± 5.44	2.16 ± 0.59	2.32 ± 3.07	1.38 ± 0.25		

Abbreviatures: P, super inductive group; Cont, control group; T, transcranial group; Comb, combined group.

### Group-by-time interaction

The statistical analysis revealed significant interactions between time and group for all measured variables. Specifically, for EMG Peak VM (μV), the interaction was significant (F = 53.75; p < 0.001; η2p = 0.786), indicating a large effect size. Similarly, for EMG Peak VL (μV), the interaction was significant (F = 67.42; p < 0.001; η2p = 0.821). EMG Peak RF (μV) also showed a significant interaction (F = 64.96; p < 0.001; η2p = 0.816). In the leg extension measures, EMG Leg Extension VM (μV) had a significant interaction (F = 93.52; p < 0.001; η2p = 0.864), while EMG Leg Extension VL (μV) showed the highest interaction (F = 140.1; p < 0.001; η2p = 0.905). EMG Leg Extension RF (μV) also demonstrated a significant interaction (F = 123.46; p < 0.001; η2p = 0.894). For dynamometry (kg), the interaction was significant (F = 21.32; p < 0.001; η2p = 0.593), indicating a moderate effect size. The CMJ (cm) showed a significant interaction (F = 15.87; p < 0.001; η2p = 0.520) (Table 3).

**Table 3:** Effects of different interventions on neuromuscular (EMG) and performance (muscle strength and CMJ) variables over time.

	T1 (Baseline) (Mean ± SD)	T2 (post 1h) (percentage change (95 CI))	T3 (post 24h) (percentage change (95 CI))	T4 (post 48h) (percentage change (95 CI))	T5 (post 72h) (percentage change (95 CI))	F	P (time x group)	η^2^p
**EMG Peak VM (μV)**								
Cont (n = 12)	522.87 ± 131.75	−54.30 (−64.72; −43.88)	−36.86 (−47.38; −26.34)	−31.13 (−41.53; −20.73)	−24.04 (−34.30; −13.78)	53.75	< 0.001	0.786
P (n = 13)	518.48 ± 97.69	−48.60 (−57.55; −39.65)	−31.40 (−40.52; −22.28)	−12.18 (−21.12; −3.24)	+6.15 (−2.68; 14.98)			
T (n = 11)	552.86 ± 139.59	−46.58 (−58.56; −34.60)	−30.99 (−42.19; −19.79)	−15.25 (−26.26; −4.24)	+5.23 (−5.80; 16.26)			
Comb (n = 12)	533.21 ± 142.51	−41.05 (−52.38; −29.72)	−29.57 (−40.70; −18.44)	−13.95 (−24.88; −3.02)	+21.92 (10.88; 32.96)			
**EMG Peak VL (μV)**								
Cont (n = 12)	428.03 ± 41.42	−62.15 (−69.09; −55.21)	−52.11 (−59.78; −44.44)	−42.42 (−50.32; −34.52)	−32.95 (−40.67; −25.23)	67.42	<0.001	0.821
P (n = 13)	456.42 ± 23.68	−62.19 (−68.46; −55.91)	−48.63 (−55.39; −41.87)	−31.59 (−38.12; −25.06)	−18.12 (−24.24; −12.00)			
T (n = 11)	447.92 ± 38.09	−50.01 (−59.02; −41.00)	−34.09 (−42.64; −25.54)	−22.99 (−31.23; −14.75)	−10.83 (−18.95; −2.71)			
Comb (n = 12)	432.53 ± 17.01	−54.03 (−58.85; −49.21)	−32.95 (−38.36; −27.54)	−8.03 (−13.17; −2.89)	+12.80 (7.02; 18.58)			
**EMG Peak RF (μV)**								
Cont (n = 12)	360.33 ± 29.74	−64.46 (−70.66; −58.25)	−54.72 (−61.28; −48.16)	−44.39 (−50.73; −38.05)	−36.27 (−42.46; −30.08)	64.96	<0.001	0.816
P (n = 13)	356.19 ± 25.05	−52.82 (−58.73; −46.90)	−41.55 (−46.73; −36.36)	−23.29 (−29.37; −17.21)	−13.71 (−18.11; −9.30)			
T (n = 11)	365.67 ± 37.93	−56.11 (−64.35; −47.87)	−37.96 (−46.33; −29.59)	−24.54 (−32.71; −16.37)	−13.35 (−20.88; −5.82)			
Comb (n = 12)	386.13 ± 13.81	−49.44 (−54.06; −44.83)	−28.68 (−33.03; −24.33)	−5.91 (−10.06; −1.76)	+16.81 (11.46; 22.16)			
**EMG Leg Extension VM (μV)**								
Cont (n = 12)	193.36 ± 54.91	−51.86 (−63.17; −40.55)	−45.03 (−56.05; −34.01)	−40.70 (−51.45; −29.95)	−32.85 (−43.33; −22.37)	93.52	<0.001	0.864
P (n = 13)	221.31 ± 78.94	−39.66 (−50.09; −29.23)	−29.03 (−38.93; −19.13)	−21.80 (−31.37; −12.23)	−0.73 (−9.79; 8.33)			
T (n = 11)	214.37 ± 65.94	−48.00 (−58.05; −37.95)	−29.25 (−38.98; −19.52)	−14.19 (−23.65; −4.73)	+16.07 (5.62; 26.52)			
Comb (n = 12)	202.65 ± 67.21	−44.44 (−54.35; −34.53)	−19.80 (−28.97; −10.63)	+9.59 (0.32; 18.86)	+46.92 (37.65; 56.19)			
**EMG Leg Extension VL (μV)**								
Cont (n = 12)	208.96 ± 31.15	−59.54 (−65.74; −53.34)	−52.36 (−58.62; −46.10)	−44.99 (−51.07; −38.91)	−39.28 (−45.24; −33.32)	140.1	<0.001	0.905
P (n = 13)	199.61 ± 27.65	−58.96 (−65.07; −52.85)	−48.07 (−54.38; −41.76)	−38.30 (−44.36; −32.24)	−22.67 (−28.49; −16.85)			
T (n = 11)	172.58 ± 34.62	−44.11 (−53.45; −34.77)	−32.53 (−41.37; −23.69)	−21.77 (−30.34; −13.20)	−9.21 (−17.57; −0.85)			
Comb (n = 12)	195.29 ± 6.10	−50.01 (−53.07; −46.95)	−28.77 (−31.60; −25.94)	−2.01 (−4.72; 0.70)	+32.12 (29.41; 34.83)			
**EMG Leg Extension RF (μV)**								
Cont (n = 12)	191.40 ± 26.85	−56.29 (−61.90; −50.68)	−51.24 (−56.89; −45.59)	−46.56 (−52.07; −41.05)	−39.66 (−44.97; −34.35)	123.46	<0.001	0.894
P (n = 13)	186.65 ± 24.80	−58.59 (−64.14; −53.04)	−49.33 (−54.98; −43.68)	−42.89 (−48.33; −37.45)	−29.21 (−34.45; −23.97)			
T (n = 11)	157.12 ± 40.36	−42.29 (−51.11; −33.47)	−32.29 (−40.82; −23.76)	−20.28 (−28.53; −12.03)	−8.62 (−16.65; −0.59)			
Comb (n = 12)	195.88 ± 7.36	−50.58 (−53.35; −47.81)	−34.09 (−36.73; −31.45)	−3.21 (−5.71; −0.71)	+31.34 (28.84; 33.84)			
**Dynamometry (kg)**								
Cont (n = 12)	46.00 ± 2.89	−19.13 (−23.60; −14.66)	−18.50 (−22.88; −14.12)	−16.09 (−20.36; −11.82)	−12.08 (−16.15; −8.01)	21.32	<0.001	0.593
P (n = 13)	43.16 ± 2.77	−21.48 (−25.87; −17.09)	−14.80 (−19.08; −10.52)	−7.76 (−11.85; −3.67)	−1.88 (−5.95; 2.19)			
T (n = 11)	49.74 ± 3.01	−23.05 (−28.02; −18.08)	−20.28 (−24.95; −15.61)	−18.18 (−22.75; −13.61)	−9.79 (−14.10; −5.48)			
Comb (n = 12)	48.93 ± 2.89	−16.25 (−20.72; −11.78)	−0.47 (−4.45; 3.51)	+6.14 (2.03; 10.25)	+16.08 (11.95; 20.21)			
**CMJ (cm)**								
Cont (n = 12)	37.33 ± 3.59	−12.03 (−16.43; −7.63)	−11.50 (−15.85; −7.15)	−8.93 (−13.15; −4.71)	−5.87 (−9.96; −1.78)	15.87	<0.001	0.520
P (n = 13)	36.35 ± 4.68	−9.38 (−13.75; −5.01)	−9.38 (−13.75; −5.01)	−5.80 (−9.98; −1.62)	−1.05 (−5.09; 3.00)			
T (n = 11)	35.99 ± 5.08	−10.58 (−15.57; −5.59)	−6.16 (−11.02; −1.30)	+0.11 (−4.67; 4.89)	+2.78 (−1.97; 7.53)			
Comb (n = 12)	41.28 ± 6.41	−9.35 (−14.05; −4.65)	−2.37 (−6.98; 2.24)	+3.41 (−1.19; 8.01)	+11.42 (6.12; 16.72)			

**Abbreviatures**: P, super inductive group; Cont, control group; T, transcranial group; Comb, combined group; EMG: surface electromyography; CMJ: Counter Movement Jump; VM, vastus medialis; VL, vastus lateralis; RF, rectus femoris; μV: micro volt; η^2^p, partial eta squared.

### Post-hoc analysis

#### EMG Peak VM.

At 24 hours post-intervention (T3), all groups demonstrated significant reductions in EMG Peak VM values. The CONT group showed the largest decline at 36.86% (p < 0.001), followed by the P group with a 31.40% reduction (p < 0.001) and the T group with a 30.99% decrease (p < 0.001). Notably, the COMB group had the smallest reduction, 29.57% (p < 0.001).

Between-group comparisons at T3 revealed no significant differences among the groups (all p > 0.0125).

At 48 hours post-intervention (T4), the Cont group’s reduction improved to 31.13% (p < 0.001), the P group improved to a 12.18% reduction (p < 0.001), the T group to 15.25% (p < 0.001), and the Comb group showed a reduction of 13.95% (p < 0.001). No significant between-group differences were detected at T4 (all p > 0.0125).

At 72 hours (T5), the Cont group exhibited further improvement with a 24.04% reduction (p < 0.001). The P group increased above baseline by 6.15% (p > 0.05), as did the T group by 5.23% (p > 0.05), while the Comb group showed the greatest improvement, with a 21.92% increase over baseline (p < 0.001). Between-group comparisons at T5 indicated that the Cont group had a significantly greater reduction compared to the T group (difference of –29.27, p = 0.003) and compared to the Comb group (–45.96, p = 0.001) (Table 3).

#### EMG Peak VL.

Significant reductions in EMG Peak VL were noted at T3 across all groups. The Cont group decreased by 52.11% (p < 0.001), followed closely by the P group with a 48.63% reduction (p < 0.001) and the T group with a 34.09% decrease (p < 0.001). The Comb group showed a smaller reduction of 32.95% (p < 0.001). At T3, the reduction in the P group was significantly smaller compared to the T group (mean difference: –14.54, p = 0.001) and the Comb group (mean difference: –15.68, p = 0.002). Additionally, the Cont group showed a significantly greater reduction than the T group (mean difference: –18.02, p = 0.001) and the Comb group (mean difference: –19.16, p = 0.001).

At T4, the Cont group improved to a 42.42% reduction (p < 0.001), the P group to 31.59% (p < 0.001), the T group to 22.99% (p < 0.001), and the Comb group to 8.03% (p < 0.001). Between‐group comparisons revealed significant differences between P and Cont (+10.83, p = 0.001), P and Comb (–23.56, p = 0.001), Cont and T (–19.43, p = 0.001), Cont and Comb (–34.39, p = 0.001), and T and Comb (–14.96, p = 0.009).

At T5, the Cont group maintained a 32.95% reduction (p < 0.001), while the P group decreased further to 18.12% (p < 0.001), the T group to 10.83% (p < 0.001), and the Comb group increased by 12.80% over baseline (p < 0.001). At T5, between‐group comparisons showed significant differences between P and Cont (+14.83, p = 0.001), P and Comb (–30.92, p = 0.001), Cont and T (–22.12, p = 0.001), Cont and Comb (–45.75, p = 0.001), and T and Comb (–23.63, p = 0.001) (Table 3).

#### EMG Peak RF.

At T3, significant declines in EMG Peak RF were observed in all groups. The Cont group exhibited the largest reduction at 54.72% (p < 0.001), followed by the P group at 41.55% (p < 0.001), and the T group at 37.96% (p < 0.001). The Comb group showed the smallest decrease at 28.68% (p < 0.001). At T3, between‐group comparisons revealed significant differences between P and Comb (–24.14, p = 0.001), Cont and T (–16.76, p = 0.001), Cont and Comb (–26.04, p = 0.001), and T and Comb (–9.28, p = 0.001).

At T4, the Cont group improved to 44.39% (p < 0.001), the P group to 23.29% (p < 0.001), the T group to 24.54% (p < 0.001), and the COMB group to 5.91% (p < 0.001). At T4, between‐group comparisons showed significant differences between Cont and Comb (–46.91, p = 0.001), Cont and T (–19.85, p = 0.001), and T and Comb (–18.63, p = 0.001); while the difference between P and Cont was not significant.

At T5, the Cont group maintained a reduction of 36.27% (p < 0.001), the P group improved to 13.70% (p < 0.001), the T group improved to 13.35% (p = 0.09), while the Comb group exceeded baseline with a 16.81% improvement (p < 0.001). At T5, between‐group comparisons revealed significant differences between Cont and P (–16.55, p = 0.001), P and Comb (–69.63, p = 0.001), Cont and T (–22.92, p = 0.001), and Cont and Comb (–53.08, p = 0.001) (Table 3).

#### EMG leg extension VM.

At T3, all groups showed significant decreases in EMG Leg Extension VM. The Cont group decreased by 45.03% (p < 0.001), the P group by 29.03% (p < 0.001), the T group by 29.25% (p < 0.001), and the Comb group by 19.80% (p < 0.001). At T3, a significant between‐group difference was observed between Cont and Comb (–25.23, p = 0.009); all other comparisons were non-significant (p > 0.0125).

At T4, the CONT group retained a 40.70% reduction (p < 0.001), while the P group improved to 21.80% (p < 0.001), the T group to 14.19% (p = 0.05), and the Comb group improved by 9.59% over baseline (p < 0.05). At T4, between‐group comparisons showed significant differences between Cont and T (–26.51, p = 0.010), and Cont and Comb (–50.29, p = 0.001).

At T5, the Cont group showed a reduction of 32.85% (p < 0.001), the P group exhibited minimal reduction at 0.73% (p > 0.05), the T group increased by 16.07% over baseline (p < 0.001), and the Comb group demonstrated the largest improvement, with a 46.92% increase over baseline (p < 0.001). At T5, between‐group comparisons revealed significant differences between P and Cont (+32.12, p = 0.002), P and Comb (–47.65, p = 0.009), Cont and T (–48.92, p = 0.001), and Cont and Comb (–79.77, p = 0.001) (Table 3).

#### EMG leg extension VL.

At T3, all groups showed significant decreases in EMG Leg Extension VL. The Cont group decreased by 52.36% (p < 0.001), the P group by 48.07% (p < 0.001), the T group by 32.53% (p < 0.001), and the Comb group by 28.77% (p < 0.001). At T3, between‐group comparisons showed that the P group differed significantly from Comb (–19.30, p = 0.003) and Cont differed significantly from Comb (–23.59, p = 0.004).

At T4, the Cont group improved to a 44.99% reduction (p < 0.001), the P group to 38.30% (p < 0.001), the T group to 21.77% (p < 0.001), and the Comb group showed a minimal reduction of 2.01% (p > 0.05). The only significant between‐group difference at T4 was between P and Comb (–36.29, p = 0.001).

At T5, the Cont group maintained a reduction of 39.28% (p < 0.001), the P group improved to 22.67% (p < 0.001), the T group improved to 9.21% (p = 0.07), and the Comb group increased by 32.12% over baseline (p < 0.001). At T5, between‐group comparisons revealed significant differences between P and Comb (–54.79, p = 0.001), Cont and T (–30.07, p = 0.001), Cont and Comb (–71.40, p = 0.001), and T and Comb (–41.33, p = 0.001) (Table 3).

#### EMG leg extension RF.

At T3, significant decreases in EMG Leg Extension RF were seen across all groups. The Cont group showed a reduction of 51.24% (p < 0.001), the P group by 49.33% (p < 0.001), the T group by 32.29% (p = 0.03), and the Comb group by 34.09% (p < 0.001). At T3, between‐group comparisons revealed significant differences between P and Comb (–15.24, p = 0.001), Cont and Comb (–17.15, p = 0.001), and T and Comb (+1.80, p = 0.002).

At T4, the Cont group improved to 46.56% (p < 0.001), the P group to 42.89% (p = 0.02), the T group to 20.28% (p = 0.08), and the Comb group showed a minimal reduction of 3.21% (p > 0.05). At T4, between‐group comparisons indicated significant differences between Cont and Comb (–43.35, p = 0.001), Cont and T (–20.28, p = 0.001), and T and Comb (–17.07, p = 0.001).

At T5, the CONT group retained a reduction of 39.66% (p < 0.001), the P group improved to 29.21% (p = 0.05), the T group improved to 8.62% (p = 0.09), and the COMB group showed an improvement of 31.34% over baseline (p < 0.001). At T5, between‐group comparisons revealed significant differences between Cont and P (–10.45, p = 0.001), P and Comb (–60.55, p = 0.001), Cont and T (–31.04, p = 0.001), and Cont and Comb (–71.00, p = 0.001) (Table 3).

#### Muscle strength.

At T3, significant reductions in muscle strength were recorded across all groups. The Cont group decreased by 18.50% (p < 0.001), the P group by 14.80% (p < 0.001), the T group by 20.28% (p = 0.02), while the Comb group showed a minimal reduction at 0.47% (p > 0.05). At T3, between‐group comparisons showed a significant difference between P and Comb (–14.33, p = 0.008).

At T4, the Cont group improved slightly to a 16.09% reduction (p = 0.003), the P group improved to 7.76% (p = 0.07), the T group improved to 18.18% (p = 0.04), and the Comb group demonstrated a 6.14% increase over baseline (p < 0.05). At T4, significant between‐group differences were observed between P and Comb (–13.90, p = 0.004), and Cont and Comb (–22.23, p = 0.002).

At T5, the Cont group showed a reduction of 12.08% (p = 0.03), the P group’s reduction was minimal at 1.88% (p > 0.05), the T group improved to 9.79% (p = 0.06), and the Comb group exhibited a significant improvement of 16.08% over baseline (p < 0.001). At T5, between‐group comparisons showed significant differences between P and Comb (–17.96, p = 0.001), and Cont and Comb (–28.16, p = 0.001) (Table 3).

#### Counter Movement Jump (CMJ).

At T3, significant reductions in CMJ performance were observed across all groups. The Cont group reduced its performance by 11.50% (p < 0.001), the P group by 9.38% (p < 0.001), the T group by 6.16% (p = 0.08), and the Comb group showed the smallest reduction at 2.37% (p > 0.05). At T3, between‐group comparisons revealed significant differences between P and Comb (–7.01, p = 0.003), and Cont and Comb (–9.13, p = 0.004).

At T4, the Cont group’s reduction improved to 8.93% (p = 0.003), the P group’s reduction decreased to 5.80% (p = 0.09), the T group showed a minimal increase of 0.11% over baseline (p > 0.05), and the Comb group showed a significant improvement with a 3.41% increase over baseline (p > 0.05). At T4, between‐group comparisons showed significant differences between Cont and COMB (–12.34, p = 0.001), and P and Comb (–9.21, p = 0.001).

At T5, the Cont group exhibited further improvement with a reduction of 5.87% (p = 0.03), the P group showed a minimal reduction of 1.05% (p > 0.05), the T group improved by 2.78% over baseline (p > 0.05), and the Comb group demonstrated the most significant enhancement, with an 11.42% increase over baseline (p < 0.001). At T5, between‐group comparisons revealed significant differences between P and Comb (–12.47, p = 0.001), Cont and Comb (–17.29, p = 0.001), and T and Comb (–8.64, p = 0.001) (Table 3).

## Discussion

The present study analyzed the effects of a combined treatment on peripheral nerves in young athletes experiencing delayed onset muscle soreness (DOMS) in the quadriceps. The combined stimulation aimed to enhance muscle activation, assessed through EMG peak readings, knee extension dynamometry, and countermovement jump (CMJ) performance.

For many years, eccentric exercise sessions leading to DOMS have been integral to training regimens designed to continually improve athletic performance. However, various therapies such as press therapy, cold water immersion, and recovery massage have not effectively mitigated post-DOMS symptoms [44]. Acknowledging the new DOMS theory proposed by Sonkodi et al. (2021), which suggests that acute axonopathy is exacerbated by muscle micro-damage and compression, this study combined transcranial electrical stimulation (TES) targeting the M1 cortex with peripheral electrical stimulation (PES) of the femoral nerve to observe male athletes’ responses to DOMS recovery [11].

This study considered a recovery time of at least 72 hours, as recommended in prior DOMS research for a complete return to baseline function [45]. Focusing on this timeframe provided deeper insights into the physiological processes involved in muscle recovery and the potential impact of therapeutic interventions on enhancing athletic performance and reducing recovery time. Each study group exhibited a decrease in performance, as shown by dynamometry and CMJ results between the T1 and T2 time points, followed by progressive recovery up to T5 (post-72 hours). This indicates that the DOMS protocol similarly affected participants across groups (Table 3).

No interaction was detected between groups for creatine kinase (CK) and lactate concentrations, suggesting uniform effects of the eccentric session across all subjects (Table 2). Quadriceps muscle activity, both during maximal voluntary contraction (peak) and leg extension, showed differences among groups shortly after the eccentric session. This suggests that acute combined electromagnetic treatment may counteract the initial decrease in muscle activity and performance observed in the early stages of DOMS.

Significant differences were found in lower limb muscle activity, particularly in quadriceps peak activity and leg extension measurements, with higher activity observed in the combined (Comb) group. These results differ from those reported by Romero-Moralda et al. (2017), who utilized foam rolling and neural mobilization, suggesting that the combined stimulation in the present study not only targeted peripheral symptoms but also enhanced central stimulation [46]. While we would have liked to measure additional parameters, we lacked the necessary equipment. Nonetheless, the observed increase in EMG values indicates functional enhancements at both subjective and objective levels.

Conversely, our findings are not directly comparable to those of Guilhem et al. (2013), who employed pulse cryotherapy for acute compression of the DOMS area. Their study did not find significant differences in peak EMG muscle activity during isometric contractions across study groups [47]. However, our results align with those of Lee et al. (2015), where Kinesiotaping led to better recovery compared to a control group at 72 hours post-DOMS [48]. The EMG dynamic activity demonstrated significant group differences. Wernbom et al. (2009) showed distinct results, as they used a blood flow restriction device aimed at increasing compression to facilitate active recovery by reducing the accumulation of metabolic waste [49]. In contrast, Bradbury-Squires et al. (2015) revealed results consistent with our findings, as they applied compressive massage to alleviate acute compression caused by the DOMS phenomenon, targeting the axonal terminations in the muscle spindle to reduce nerve excitability [50].

The significant differences between study groups in both peak and active EMG could be linked to the DOMS theory proposed by Sonkodi et al. (2021), suggesting an acute axonopathy. This theory stablishes that DOMS may originate from acute axonopathy, potentially explaining why muscle function is impaired during the DOMS process. Since fatigue is not solely a reflection of glycogen depletion and is influenced by various factors, our analysis aimed to include the assessment of neural feedback from fatigued muscles [3,11,51]. This approach provides a deeper understanding of muscle and nerve interactions during recovery phases, indicating that simultaneous stimulation of both central and peripheral nervous systems may play a crucial role in accelerating recovery and enhancing muscle performance post-exercise. The implications could be significant in sports medicine, offering a new perspective on the treatment and management of DOMS and related muscular stress injuries [11].

CK concentration levels were analyzed to corroborate subjective data, ensuring that all participants experienced a DOMS process. Thus, CK concentration serves not as a dependent outcome of the study but as an intervention outcome to validate the accuracy of the DOMS protocol employed between T1 and T2.

The peak isometric force measured through dynamometry revealed differences, with the Comb group generating greater force. This aligns with findings by Rezaei et al. (2014), which indicated that Kinesiotaping could enhance muscle fiber activity and improve muscle performance [52]. A positive correlation between force dynamometry and EMG results suggests higher muscle activity at 72 hours post-DOMS in the Comb group. However, the variability in results observed by Keriven et al. (2023) may be attributed to their exclusive use of peripheral stimulation, which primarily affects the peripheral nerve. This stimulation activates ascending pathways that convey messages to the central nervous system, potentially increasing blood flow to somatosensory areas and altering pain perception related to the stimulation zone [14].

Decades of scientific research have demonstrated that muscles undergoing delayed onset muscle soreness (DOMS), or injury exhibit abnormal responses. Both the central and peripheral nervous systems experience alterations that may impact the function of individual muscles or groups of muscles. In the study by Lin et al. (2021), it was observed that transcranial magnetic stimulation (TMS) could be employed to assess whether peripheral nerves are appropriately executing their functions by receiving activation signals, similar to the findings in the present study, particularly with respect to the electromyographic (EMG) results [53]. Furthermore, research by Lang et al. (2007) has shown that peripheral muscle stimulation (PMS) has the potential to potentiate synaptic activity, as evidenced by the increase in muscle strength observed in the current study [54]. In the work carried by Kweon et al. (2023) it has been observed that an application of a combined treatment of electromagnetic stimulation could increase nerve function which is a similar finding as in our study [55].

In this study, the combined treatment led to normalization of both central and peripheral nervous systems, indicating that differences observed in the Comb group may suggest that muscle fatigue could be linked to dysfunction in both systems. Our treatment approach successfully enhanced this function.

Lower limb performance, assessed through CMJ, also demonstrated differences between groups, with the Comb group achieving better results than others. Our findings contrast with those of Dominguez-Balmaseda et al. (2020), who conducted a similar DOMS protocol but analyzed recovery using a different intervention involving zingiber supplementation [56]. These findings support the notion that direct stimulation of the peripheral nerve, combined with central nervous system stimulation, contributes not only to improved recovery but also to enhanced performance in motor tasks. Recent studies have demonstrated that combined stimulation could reduce DOMS symptoms such as pain and pressure thresholds [24]. Additionally, results from Romero-Moraleda et al. (2019) using vibrative massage to alleviate DOMS-related performance decrements also reported improved CMJ outcomes [57].

### Practical implications

The present study could serve as a valuable tool in the field of recovery, providing athletes and coaches with a comprehensive explanation of the theory and origins of DOMS. Given that athletes are required to compete more frequently each year, the practical implications of this study could benefit many professional athletes. Additionally, it introduces a promising new treatment that can be implemented from the early stages of recovery. Since it is both functional and time-efficient, it can be seamlessly integrated into a recovery program during the competitive season.

In summary, the combined transcranial and peripheral electromagnetic stimulation demonstrated positive effects on muscle activity, force development, and performance recovery in athletes experiencing DOMS. These findings suggest the potential of this combined approach to enhance recovery and performance, providing a novel perspective in the field of DOMS management.

### Limitations of the study and future research lines

The present study has some limitations to consider. First, it is crucial to acknowledge that the results obtained may not be applicable to less active populations or women. In addition, future studies should include an analysis of the knee range of motion to determine the treatment’s influence on parameters such as oedema or inflammation, which may affect joint mobility [44].

Concerning a possible placebo effect, in the present study the control group had both devices positioned in the same manner as in the treatment group, but with the devices turned off. These stimulations were performed below the threshold, which was previously calculated based on 5–10 contractions at 50 microvolts for each participant. Afterward, participants were unable to distinguish whether they were in the stimulation group or not. This could lead to a placebo effect and could have influenced the results of the control groupo [58].

Exploring the interaction with other treatments, such as supplementation for DOMS recovery might provide valuable insights [59]. Physical parameters or hydric levels could have influenced the results of the present study it could be relevant to add a measurement of those in future work would allowed to isolate the results. Also, it might be interested to begin a recovery pattern study weeks before a study like the present in an aim to identified and individualized the recovery pattern for each subject.

As a novel approach in the field of recovery treatments, future research could focus on applying the present combined treatment over an entire season in a group of athletes. This would allow for the analysis of various parameters, providing a long-term perspective on the effects of the treatment. Such an approach would extend beyond the current study, which was limited to 72 hours post-DOMS induction. Another approach could explore the synergy of the treatment and its effects when combined with conventional recovery methods. This would help determine whether such a combination enhances or diminishes the potential benefits of the applied therapies.

Finally, in future works, parameters such as heart rate variability or central fatigue induced by exercise should be observed, as they play a notable role in post-exercise recovery.

## Conclusions

The combined treatment demonstrated positive effects on muscle activity and lower limb performance in young athletes. This study indicates that a combined treatment approach could shed light on the generation of DOMS based on an acute axonopathy, strengthening Sonkodi’s theory. These findings suggest that the DOMS theory could be validated by this work, and a combination of peripheral and central stimulation has the potential to reduce associated symptoms while enhancing performance and muscle activity, without altering physiological adaptations natural of DOMS. These novel findings warrant further research, especially in diverse study populations.

## Supporting information

S1 Study protocol, Spanish version. (DOCX)

S2 Study protocol, English version. (DOCX)

S3 Dataset. (CSV)
