# Supplementary material for: Neurophysiological outcomes of combined transcranial and peripheral electromagnetic stimulation on DOMS among young athletes: A randomized controlled trial
Source: PLoS One. 2025 Jul 8;20(7):e0312960. doi: 10.1371/journal.pone.0312960 (PMC12237059; doi:10.1371/journal.pone.0312960)
Supplement: S1 — (DOCX) [file pone.0312960.s001.docx]

**PROTOCOLO DE ESTUDIO – ENSAYO DE RECUPERACIÓN DE DOMS**

**Diseño y entorno del estudio**

Se realizará un ensayo controlado, aleatorizado y doble ciego en el que participarán jóvenes atletas. Se seguirán las directrices éticas de Helsinki y todos los participantes, tras recibir una explicación presencial del procedimiento del estudio, completarán un formulario de consentimiento informado antes de comenzar el estudio.

Los participantes se distribuirán en uno de cuatro grupos distintos: el grupo de control (Cont), que no recibió ninguna intervención; el grupo de superinducción (P); el grupo transcraneal (T); y el grupo de combinación de estimulación (Comb). Se designará un lugar neutral para administrar los tratamientos y los investigadores asignados a las estaciones de tratamiento participarán exclusivamente en la administración de las intervenciones.

**Cálculo del tamaño de la muestra**

El cálculo del tamaño de la muestra para el estudio se ha realizado utilizando el software G*Power. Se seleccionó una prueba F para un ANOVA con medidas repetidas, teniendo en cuenta la interacción intra-intermedia. Los parámetros de entrada incluyeron un tamaño del efecto medio f=0,20, un nivel de significación de 0,05, una potencia de 0,8, cuatro grupos, cinco mediciones y una corrección de no esfericidad de 1. Los resultados indicaron que se requiere un tamaño de muestra total de 48 participantes para lograr la potencia estadística suficiente. Para tener en cuenta las posibles pérdidas del 10%, el tamaño de la muestra se aumentará a 52 participantes (13 por grupo) para compensar los posibles abandonos.

**Participantes**

La investigación se realizará con participantes de una universidad, seleccionados en base a criterios específicos de inclusión y exclusión para asegurar la relevancia de los resultados del estudio. Los esfuerzos de reclutamiento se implementarán a través de canales de comunicación y anuncios colocados estratégicamente dentro de la Facultad de Ciencias del Deporte de la universidad.

Los criterios de inclusión serán varones de entre 18 y 35 años de edad, que realicen actividad física de forma regular al menos tres veces por semana durante un mínimo de un año y que no presenten hipersensibilidad en las zonas a tratar con estimulación periférica. Los criterios de exclusión serán no haber sido diagnosticado de ninguna enfermedad crónica, no haber sufrido una lesión musculoesquelética en la extremidad inferior en los seis meses anteriores y no ser fumador.

**Aleatorización**

La aleatorización de los grupos se realizará utilizando la función de aleatorización de Microsoft Office Excel (Microsoft Corporation, Redmond, Washington, EE. UU.). Los participantes serán asignados a uno de los cuatro grupos de estudio descritos anteriormente. Los examinadores y el analista de datos no conocerán la asignación de grupo de los participantes durante el estudio, ya que se diseñará una sala especial para que los participantes reciban los tratamientos.

**Procedimiento**

La metodología del estudio implicará una serie estructurada de cinco sesiones de evaluación para cada participante. Una semana antes del comienzo de la primera evaluación se realizará una sesión de familiarización para que los participantes se familiaricen con los procedimientos y el equipo.

La sesión de evaluación (T1) será integral y abarcará una variedad de mediciones fisiológicas y biomecánicas, como la recolección de niveles de creatina quinasa (CK), concentración de lactato en sangre, rendimiento en el Counter Movement Jump (CMJ), dinamometría del cuádriceps, electromiografía de superficie (EMG) del cuádriceps y datos antropométricos.

Se programarán sesiones posteriores a intervalos específicos después del ejercicio, es decir, 1 hora (T2), 24 horas (T3), 48 horas (T4) y 72 horas (T5) después de la inducción del daño muscular, para realizar un seguimiento de la progresión y la recuperación a lo largo del tiempo.

La evaluación del daño muscular se centrará principalmente en el análisis de las concentraciones de CK y lactato en sangre, obtenidas a partir de muestras de sangre obtenidas mediante punción con aguja y que se analizarán mediante análisis electroforético (Lactate Scout Pro, Musimedic SL Donostia, España). Para garantizar la precisión de estas mediciones enzimáticas, se indicará a los participantes que se abstengan de realizar cualquier actividad física durante al menos dos días antes del estudio, antes de la línea de tiempo inicial (tiempo de estudio T1). Los participantes podrían reanudar una actividad física normal después de completar el primer día (incluido el tiempo de estudio T1 y T2).

**Intervención**

***Protocolo de ejercicios excéntricos***

La sesión de ejercicios se diseñará en tres fases distintas.

1. Calentamiento general: La fase inicial incluirá un calentamiento, centrado en mejorar la movilidad articular de las extremidades inferiores y realizar ejercicios de fuerza con el peso corporal. Esta fase preparatoria preparará a los atletas para los ejercicios posteriores destinados a inducir DOMS.

2. Ejercicios de intervención: Los participantes realizarán una serie de tres ejercicios. La piedra angular de esta fase será el ejercicio de sentadilla controlado por encoder. La ejecución de la sentadilla se cuantificará utilizando un acelerómetro lineal, calibrado para medir el 60% de la repetición máxima (1RM) del participante (González-Badillo et al., 2011).

3. Rutina de entrenamiento excéntrico: La fase final comprenderá los siguientes tres ejercicios:

a) Sentadilla hacia adelante, 10 series de 10 repeticiones al 60% del 1RM del participante, valor establecido durante la sesión de entrenamiento previa al estudio.

b) Sentadilla búlgara, 3 series de 10 repeticiones para cada pierna, con opción de añadir 5 o 10 kg más de peso.

c) Viga hacia adelante (Split), 3 series de 10 repeticiones con cada pierna, con posibilidad de añadir de 5 a 10 kg de peso.

***Protocolo de estimulación electromagnética periférica y transcraneal en el estudio***

Grupo de control (cont.): la máquina de estimulación electromagnética se colocará de la misma manera que en los grupos de tratamiento activos. Sin embargo, la máquina se apagará y se reproducirán sonidos pregrabados de su funcionamiento durante la sesión de tratamiento.

Grupo Super Inductivo (P): Se administrará el tratamiento PES siguiendo el protocolo de Potenciación a Largo Plazo. Este protocolo constará de cinco estimulaciones a 100 Hz, cada una de ellas de 5 segundos de duración, intercaladas con intervalos de descanso de 55 segundos..El tiempo total de estimulación para este grupo será de 10 minutos.

Grupo de estimulación transcraneal (T): este grupo recibirá un tratamiento TES que implica 2000 pulsos administrados durante una duración mínima de 20 minutos, dirigidos al área cortical M1.

Grupo de estimulación combinada (Comb): Los participantes de este grupo recibirán una combinación de tratamientos PES y TES, y el tiempo total de estimulación se extenderá a 30 minutos.

En todos los grupos, el tratamiento se iniciará una hora después de la sesión de ejercicio excéntrico, alineándose con el inicio de la fatiga, momento T2 en el estudio.

Los tratamientos TES y PES se administrarán utilizando un estimulador magnético MagRex equipado con una bobina en forma de anillo / bobina en forma de 8 (MR Inc., República de Corea, http://www.mrev.co.kr).

**Medida de resultado primaria:Electromiografía de superficie**

En esta investigación se empleará el sistema EMG mDurance® (mDurance Solutions SL, Granada, España). Este sistema EMG de superficie portátil (sEMG) integra tres componentes: sensores, computación móvil y un marco de análisis de datos basado en la nube.

1. Primer sensor: evaluación del recto femoral (RF): los participantes se colocarán en una camilla, con las rodillas ligeramente flexionadas y el tronco inclinado hacia atrás. Se colocarán electrodos en el punto medio entre la espina ilíaca anterosuperior (EIA) y la parte superior de la rótula. El electrodo de tierra se colocará en la rótula.

2. Segundo sensor: registro de actividad del vasto lateral (VL) y del vasto medial (VM): en el caso del VE, los electrodos se colocarán a dos tercios de la distancia a lo largo de la línea que va desde el EIAS hasta la parte lateral de la rótula. En el caso del VM, los electrodos se colocarán al 80 % de la distancia desde el EIAS hasta el borde anterior del ligamento lateral interno.

La actividad muscular superficial se capturará utilizando dos sensores bipolares proporcionados por Shimmer Research Ltd, Dublín, Irlanda. El registro y la transmisión de datos se facilitarán mediante la aplicación móvil mDurance®, instalada en una tableta Android Galaxy A7 (ZtotopCase, Suwon, Corea del Sur)..

**Medidas de resultados secundarios:**

***Dinamometría***

El estudio utilizará dinamometría de fuerza, utilizando un dispositivo ActiveForce 2 (Activbody, San Diego, CA). La fuerza isométrica máxima del cuádriceps se medirá durante la extensión de la rodilla mientras el participante está sentado, con la rodilla colocada en un ángulo de 90 grados. Se aplicará una correa limitadora de movimiento en el tercio medio de la pierna. Los participantes deberán mantener la contracción durante 5 segundos, repitiendo el proceso dos veces con un intervalo de descanso de 5 minutos entre las contracciones.

***Salto con contramovimiento***

Los participantes realizarán el CMJ con las manos en las caderas, realizando una flexión de rodilla seguida de una extensión rápida para alcanzar la altura máxima de salto. El CMJ se grabará utilizando la aplicación validada My Jump 2, capturando cada salto en cámara lenta a 240 cuadros por segundo con un iPad Pro 10. La tableta se colocará de manera uniforme para cada grabación para garantizar una captura de datos estable y confiable.

**Análisis estadístico**

Se utilizará el programa SPSS v.29 (IBM, Armonk, NY, EE. UU.) para realizar el análisis estadístico. Se evaluará la distribución normal de los datos mediante histogramas y la prueba de Shapiro-Wilk. Se considerará que las variables con un valor p inferior a <0,05 tienen una distribución no normal, mientras que las variables con un valor p superior a 0,05 se considerarán que tienen una distribución normal. Para describir la muestra, se informará la media y la desviación estándar para las variables de distribución normal, mientras que se informará la mediana y el rango intercuartil para las variables de distribución no normal. Se utilizarán pruebas t independientes o pruebas U de Mann-Whitney para comparar las medias entre los grupos al inicio del estudio para las variables cuantitativas. Cuando se cumplan los supuestos, se realizará un análisis de varianza (ANOVA) mixto 5x4 (5 mediciones x 4 grupos), con corrección para comparaciones múltiples mediante la prueba de Bonferroni. El tamaño del efecto, expresado como eta cuadrado parcial (ηp2​), se puede clasificar en tres rangos: un efecto pequeño (0,01), un efecto medio (0,06) y un efecto grande (0,14).
