# Supplementary material for: Neurophysiological outcomes of combined transcranial and peripheral electromagnetic stimulation on DOMS among young athletes: A randomized controlled trial
Source: PLoS One. 2025 Jul 8;20(7):e0312960. doi: 10.1371/journal.pone.0312960 (PMC12237059; doi:10.1371/journal.pone.0312960)
Supplement: S2 — (DOCX) [file pone.0312960.s002.docx]

**STUDY PROTOCOL – DOMS RECOVERY TRIAL**

**Study Design and Setting**

A randomized, controlled, double-blind trial involving young athletes will be performed. The Helsinki ethical guidelines will be followed, and all the participants after receiving a presential explanation of the study’s procedure will complete an informed consent form before starting the study.

Participants will be allocated into one of four distinct groups: the Control group (Cont), which received no intervention; the Super Induction group (P); the Transcranial group (T); and the Combination of Stimulation group (Comb). A neutral location will be designated for administering the treatments, and researchers assigned to the treatment stations will be exclusively involved in administering the interventions.

**Sample size calculation**

The sample size calculation for the study has been performed using G*Power Software. An F-test was selected for an ANOVA with repeated measures, accounting for within-between interaction. Input parameters included a medium effect size f=0.20, a significance level of 0.05, a power of 0.8, four groups, five measurements, and a nonsphericity correction of 1. The results indicated that a total sample size of 48 participants is required to achieve the sufficient statistical power. To account for potential losses of 10%, the sample size will be increased to 52 participants (13 per group) to compensate for possible dropouts.

**Participants**

The research will be conducted with participants from a university, selected based on specific inclusion and exclusion criteria to ensure the relevance of the study results. Recruitment efforts will be implemented through communication channels and advertisements strategically placed within the Faculty of Sports Sciences of the university.

The inclusion criteria will be males aged between 18 and 35 years; engaged in regular physical activity at least three times a week for a minimum duration of one year; and exhibiting no hypersensitivity in areas to be treated with peripheral stimulation. Exclusion criteria will be not to have been diagnosed with any chronic disease; not to have sustained a musculoskeletal injury to the lower extremity within the preceding six months; and not to be smokers.

**Randomization**

Randomization of groups will be performed using the randomization function of Microsoft Office Excel (Microsoft Corporation, Redmond, Washington, USA). Participants will be assigned to one of four study groups as described previously. The examiners and the data analyst will be blinded to the participant’s group allocation during the study as a special room will be designed for the participants to receive the treatments.

**Procedure**

The study's methodology will involve a structured series of five assessment sessions for each participant. A familiarization session will be conducted one week prior to the commencement of the first assessment to acquaint participants with the procedures and equipment.

The assessment session (T1) will be comprehensive, encompassing a range of physiological and biomechanical measurements, such as the collection of creatine kinase (CK) levels, blood lactate concentration, performance in the Counter Movement Jump (CMJ), dynamometry of the quadriceps, surface electromyography (EMG) of the quadriceps, and anthropometric data.

Subsequent sessions will be scheduled at specific intervals post-exercise - namely 1 hour (T2), 24 hours (T3), 48 hours (T4), and 72 hours (T5) after the induction of muscle damage - to track the progression and recovery over time.

The evaluation of muscle damage will be primarily focused on the analysis of blood CK and lactate concentrations, obtained from a pin prick blood samples and will be analyzed using electrophoretic analysis (Lactate Scout Pro, Musimedic S.L Donostia, Spain). To ensure the accuracy of these enzyme measurements, participants will be instructed to abstain from any physical activity for at least two days prior to the study, before the baseline timeline (T1 study time). The participants could resume a normal physical activity after completing the first day (including T1 and T2 study time).

**Intervention**

***Eccentric exercise protocol***

The exercise session will be designed in three distinct phases.

1. General Warm-Up: The initial phase will involve a warm-up, focusing on enhancing joint mobility in the lower limbs and engaging in bodyweight strength exercises. This preparatory phase will condition the athletes for the subsequent exercises aimed at inducing DOMS.

2. Intervention Exercises: Participants will engage in a series of three exercises. The cornerstone of this phase will be the encoder-controlled squat exercise. Execution of the squat will be quantified using a linear accelerometer, calibrated to measure 60% participant's one-repetition maximum (1-RM) (González-Badillo et al., 2011).

3. Eccentric Workout Routine: The final phase will encompass the following three exercises:

a) Squat Forward, 10 sets of 10 repetitions at 60% of the participant’s 1-RM, a value established during the pre-study workout session.

b) Bulgarian Squat, 3 sets of 10 repetitions for each leg, with an option to add an additional 5 or 10 kg of weight.

c) Forward Beam (Split), 3 sets of 10 repetitions on each leg, with the possibility of adding 5 to 10 kg of weight.

***Protocol for Transcranial and Peripheral Electromagnetic Stimulation in the Study***

Control (Cont) Group: The electromagnetic stimulation machine will be positioned identically as in the active treatment groups. However, the machine will be turned off, and pre-recorded sounds of the machine's operation will be playing during the treatment session.

Super Inductive (P) Group: The PES treatment will be administered, following the Long-Term Potentiation protocol. This protocol will entail five stimulations at 100 Hz, each lasting for 5 seconds, interspersed with 55-second rest intervals. The total stimulation time for this group will be 10 minutes.

Transcranial Stimulation (T) Group: This group will receive TES treatment involving 2000 pulses administered over a minimum duration of 20 minutes, targeting the cortical area M1.

Combined Stimulation (Comb) Group: Participants in this group will receive a combination of both PES and TES treatments, with the total stimulation time extending to 30 minutes.

In all groups, the treatment will start one hour post the eccentric exercise session, aligning with the onset of fatigue, time T2 in the study.

The TES and PES treatments will be administered using a MagRex magnetic stimulator equipped with a ring-shaped coil / 8-shaped coil (MR Inc., Republic of Korea, http://www.mrev.co.kr).

**Primary Outcome Measure: Surface Electromyography**

In this research, the mDurance® EMG system (mDurance Solutions SL, Granada, Spain) will be employed. This portable surface EMG (sEMG) system integrates three components: sensors, mobile computing, and a cloud-based data analysis framework.

1. First Sensor – Rectus Femoris (RF) Evaluation: Participants will be positioned on a stretcher, with knees slightly flexed and the trunk leaning backwards. Electrodes will be placed at the midpoint between the anterior superior iliac spine (AIS) and the top of the patella. The ground electrode will be positioned on the patella.

2. Second Sensor – Vastus Lateralis (VL) and Vastus Medialis (VM) Activity Recording: For the VE, electrodes will be positioned at two-thirds of the distance along the line from the EIAS to the lateral part of the patella. For the VM, electrodes will be placed at 80% of the distance from the EIAS to the anterior border of the internal lateral ligament.

Surface muscle activity will be captured using two bipolar sensors provided by Shimmer Research Ltd, Dublin, Ireland. Data recording and transmission will be facilitated by the mDurance® mobile application, installed on a Galaxy A7 Android Tablet (ZtotopCase, Suwon, South Korea).

**Secondary Outcome Measures:**

***Dynamometry***

The study will use strength dynamometry, using an ActiveForce 2 device (Activbody, San Diego, CA). Maximal isometric strength of the quadriceps will be measured during knee extension while the participant is seated, with the knee positioned at a 90-degree angle. A motion-limiting strap will be applied to the middle third of the leg. The participants will be required to sustain the contraction for 5 seconds, repeating the process twice with a 5-minute rest interval between contraction.

***Counter Movement Jump***

Participants will perform the CMJ with their hands on their hips, executing a knee flexion followed by a rapid extension to achieve maximum jump height. The CMJ will be recorded using the validated My Jump 2 application, capturing each jump in slow motion at 240 frames per second with an iPad Pro 10. The tablet will be positioned consistently for each recording to ensure stable and reliable data capture.

**Statistical analysis**

SPSS v.29 (IBM, Armonk, NY, USA) will be used to perform statistical analysis. Normal distribution of data will be assessed using histograms and the Shapiro-Wilk test. Variables with a p-value less than <0.05 will be considered to have a non-normal distribution, while variables with a p-value greater than 0.05 will be considered to have a normal distribution. To describe the sample, the mean and standard deviation will be reported for normally distributed variables, while the median and interquartile range will be reported for non-normally distributed variables. Independent t tests or Mann-Whitney U tests will be used to compare means between groups at baseline for quantitative variables. When the assumptions are met, a 5x4 (5 measurements x 4 groups) mixed analysis of variance (ANOVA) will be performed, with correction for multiple comparisons using the Bonferroni test. The effect size, expressed as partial eta squared (ηp2​), can be categorized into three ranges: a small effect (0.01), a medium effect (0.06), and a large effect (0.14).
